# The nematicidal potential of *Moringa oleifera* extracts and rhizobacteria against *Meloidogyne incognita* in tomato

**DOI:** 10.3389/fpls.2025.1562074

**Published:** 2025-05-13

**Authors:** Wajahat Azeem, Tariq Mukhtar, Muhammad Inam-ul-Haq, Muhammad Azam Khan, Muhammad Suhail Ibrahim, Ahmad Hassan, Homan Regmi, Larry W. Duncan

**Affiliations:** ^1^ Department of Plant Pathology, Pir Mehr Ali Shah Arid Agriculture University, Rawalpindi, Pakistan; ^2^ Citrus Research and Education Center (CREC), University of Florida, Lake Alfred, FL, United States; ^3^ Department of Horticulture, Pir Mehr Ali Shah Arid Agriculture University, Rawalpindi, Pakistan; ^4^ Institute of Food and Nutritional Sciences, Pir Mehr Ali Shah Arid Agriculture University, Rawalpindi, Pakistan; ^5^ School of Biochemistry and Biotechnology, University of the Punjab, Lahore, Pakistan

**Keywords:** root-knot nematodes, biological control, *Meloidogyne incognita*, *Moringa oleifera*, rhizobacteria, bacterial VOCs, FTIR, GC-MS

## Abstract

Here we report the effects of aqueous extracts of the plant *Moringa oleifera* and rhizobacterial strains, *Bacillus australimaris* (BA-LWD73) and *B. thuringiensis* (BT-WAG41), applied singly and in combination, on tomato growth and *Meloidogyne incognita* infection. Plant height, root length, shoot and root biomass (fresh and dry), were significantly increased by most treatments compared to the control. The combined treatment of BA-LWD73 + *M. oleifera* produced the highest plant height (96.94 cm) and root length (30.48 cm). The highest shoot biomass was observed in BA-LWD73 alone treatment while root biomass was generally lower in all treatments than in the control. *M. incognita* induced, root gall rating, number of eggs per root system, second stage juveniles (J2), and reproduction rate, were significantly reduced in treatments involving *M. oleifera* and BA-LWD73, either alone or in combination. The lowest root gall rating (2.67) and J2 count (680) were observed in *B. australimaris* LWD73 + *M. oleifera* treatment. FTIR analysis of *M. oleifera* extract indicated the presence of functional groups such as hydroxyl, C=C, S=O, and C–O, suggesting bioactive potential. GC-MS analysis revealed six phytocompounds, with 5-Hydroxymethylfurfural (71.76%) as the dominant component, all known for antimicrobial and nematicidal activities. Moreover, volatile organic compounds from BA-LWD73 included 1H-Indole (87.46%) and 2-Nonanone (6.54%), known for their nematicidal properties. These findings highlight the potential of rhizobacteria and plant extracts in promoting tomato growth and suppressing *M. incognita* infection.

## 1 Introduction

Root-knot nematodes (*Meloidogyne* spp.) have a devastating impact on vegetable crops worldwide, causing significant yield losses and compromising overall plant health (Ornat et al., 2001; Haq et al., 2022; Yaseen et al., 2023; Shahid et al., 2024). These polyphagous nematodes have an extensive host range, infecting over 3,000 plant species (Kayani et al., 2013; Kayani and Mukhtar, 2018; Hussain and Mukhtar, 2019; Khairy et al., 2021; Saeed et al., 2021, 2023). Upon infection, they induce the formation of root galls, which disrupt normal root functions and impair the ability of plants to efficiently absorb water and nutrients from the soil (Karssen and Moens, 2006; Nazir et al., 2019; Khan et al., 2019; Saeed and Mukhtar, 2024). This leads to stunted growth and reduced crop yields (Mukhtar et al., 2013a; Abbas et al., 2024). Moreover, root-knot nematode infections often predispose plants to secondary infections by soil-borne pathogens, exacerbating damage and further diminishing plant health (Asghar et al., 2020; Ahmed et al., 2021; Yaseen et al., 2024; 2025).

The severity of the damage caused by root-knot nematodes depends on various factors, including environmental conditions, nematode population density, species virulence, and the susceptibility of the host plant (Tariq-Khan et al., 2017, 2020, 2025). Managing these nematodes is particularly challenging due to their high reproductive potential and remarkable adaptability (Hussain et al., 2011; Ayub et al., 2024). Although chemical nematicides have proven effective in controlling root-knot nematodes, their adverse environmental impacts have driven a shift toward developing alternative, management strategies (Yaseen and Mukhtar, 2024).

Tomato (*Lycopersicon esculentum* L.) is a major crop in Pakistan, cultivated on 68,863 hectares with an average yield of 11.08 t/ha, significantly lower than in China (59.2 t/ha), Turkey (79.3 t/ha), and India (25.1 t/ha) (FAO, 2021). The crop is vulnerable to various diseases caused by fungal, bacterial, viral, and nematode infections, leading to substantial yield losses (Aslam et al., 2017; Hassan et al., 2024; Iqbal et al., 2024; Mustafa et al., 2024; Vagelas, 2024).

Root-knot nematodes pose a significant threat, causing yield losses of up to 29%. In India, nematode-related losses reach $21.7 million, and the Mediterranean region reports losses of 62% (Kumar et al., 2020; Fullana et al., 2023). Pakistan also faces considerable economic damage from root-knot nematodes resulting in reduced plant vigor, chlorosis, stunted growth, and potential crop failure (Oka et al., 2000). Infected plants develop weak root systems, making them easily uprooted (Anwar and Javed, 2010).

The use of biological control agents, primarily bacteria and fungi, presents a promising alternative approach to suppressing nematode populations. Similarly, plant-based natural products have proven effective against various pathogens including root-knot nematodes (Aslam et al., 2024; Atiq et al., 2024; Manzoor et al., 2024; Taha et al., 2024). Incorporating macerated leaves of *Cannabis sativa* and *Azadirachta indica* into the soil significantly reduced *M. javanica* populations and infection severity in plants in a dose-dependent manner (Kayani et al., 2012; Mukhtar et al., 2013b). Likewise, the combined application of dried neem leaves and *Trichoderma harzianum* on tomato plants increased reductions in root galls, egg mass production, and egg hatching compared to their individual applications. This synergistic effect also enhanced plant height and fresh shoot weight, with outcomes strongly influenced by dosage and exposure time (Azeem et al., 2021).

Alternative nematode management strategies include gene editing, natural nematicides (bacteria, fungi, botanicals), and cultural practices, reducing reliance on chemical controls (Afzal and Mukhtar, 2024). Plant growth-promoting rhizobacteria (PGPR) are essential for enhancing plant growth and mitigating stress through increased mineral availability, phytohormone production, alleviating heavy metal stress, and biocontrol of pathogens leading to better nutrient uptake, growth, and crop yields (Khandagale et al., 2024; Zhang et al., 2024). For sustainable rice and sugarcane production, bacteria from the genera *Bacillus*, *Rhizobium*, *Comamonas*, *Cyanobacteria*, *Nodosilinea*, *Levinella*, and *Pseudomonas* effectively produce nitrogen and solubilize essential nutrients (Khandagale et al., 2024). A consortium of *B. cereus* AR156, *B. subtilis* SM21, and *Serratia* sp. XY21 has been shown to reduce root-knot disease severity while enhancing cucumber yield and fruit quality (Zhang et al., 2024).

*Moringa oleifera* has shown promise as a natural nematicide, with its bioactive compounds disrupting nematode life cycles and improving plant vigor and yield. When combined with beneficial rhizobacteria, *M. oleifera* enhances nematode suppression, offering an eco-friendly approach to nematode management (Geldenhuys, 2023; Aminisarteshnizi, 2024).

The present study, therefore, aims to evaluate the nematicidal potential of *M. oleifera*, both alone and in combination with PGPR, to identify effective, alternative solutions for managing *Meloidogyne incognita* in tomato.

## 2 Materials and methods

### 2.1 Inoculum preparation of nematode

*Meloidogyne incognita* culture, maintained in the Citrus Research and Education Center (CREC), University of Florida, USA, was initiated from a single egg mass on a susceptible tomato host (cv. HM1824) and identified based on perineal pattern morphology (Taylor and Netscher, 1974). Infected root fragments were shaken in 0.5% sodium hypochlorite (NaOCl) to release eggs. The suspension was filtered through a 200-mesh sieve to remove debris and a 500-mesh sieve to collect eggs. The eggs were rinsed three times with tap water to eliminate residual bleach. Freshly hatched second-stage juveniles (J2) were obtained by incubating eggs at 25°C for 48 h and used as inoculum (Kayani et al., 2018).

### 2.2 Preparation of aqueous extracts of *M. oleifera*

Fresh *M. oleifera* leaves were washed, blended in sterile water, and left for 12 h. The mixture was filtered sequentially through muslin cloth, Whatman No. 1 filter paper, and a Millipore filter. Different extract concentrations (0 to 100%) were prepared by dilution with distilled water (Mukhtar et al., 2013c, d).

### 2.3 Isolation and purification of rhizobacteria

Rhizosphere soil from tomato plants was collected, and rhizobacteria were isolated via serial dilution (Shahzaman et al., 2015; Aziz et al., 2024; Aslam et al., 2025). Dilutions (10^-5^, 10^-6^ and 10^-7^) were plated on nutrient agar (NA) and incubated at 26 ± 2°C. After 24 h, individual colonies were purified by streaking (Aslam and Mukhtar, 2023a, b, 2024).

### 2.4 Molecular characterization of rhizobacterial isolates

Genomic DNA was extracted using a commercial kit. The 16S rRNA gene was amplified via PCR with universal primers 27F and 1492R (Kumar et al., 2018). PCR conditions: initial denaturation (94°C, 3 min); 35 cycles of 94°C (40 s), 60°C (50 s), 72°C (1 min); final extension (72°C, 10 min). Amplified DNA was visualized, purified, quantified (NanoDrop), and sequenced (Eurofins Genomics, USA).

### 2.5 Sub-culturing and preparation of rhizobacterial suspensions

Identified strains were cultured on NA, transferred to Luria Bertani broth, and incubated at 25°C (48 h, rotary shaker). Cell suspensions were adjusted to OD_600_ = 1.0 (10^9^ CFU/mL), centrifuged (5000 rpm, 15 min, 4°C), washed, and resuspended in sterile water (Nikoo et al., 2014).

### 2.6 Effect of rhizobacteria and plant extracts on plant growth and *M. incognita* infection

Tomato plants (21-day-old) were inoculated with 5000 J2 *M. incognita* (5 mL suspension). After 48 h, 50 mL of rhizobacterial suspension (10^9^ CFU/mL) or *M. oleifera* extract was applied via soil drench (Mukhtar et al., 2013b). Controls received water. Six replicates per treatment were maintained (**Table 1**).

**Table 1:** Treatments involving rhizobacteria and moringa leaf extracts for assessing plant growth and *Meloidogyne incognita* infection.

Treatment	Description
T1	*M. oleifera + Meloidogyne incognita*
T2	*Bacillus australimaris LWD73 + M. incognita*
T3	*B. australimaris LWD73 + M. oleifera + M. incognita*
T4	*B. cereus* HR001*+ M. incognita*
T5	*B. cereus* HR001 *+ M. oleifera + M. incognita*
T6	*B. thuringiensis* WAG41 *+ M. incognita*
T7	*B. thuringiensis* WAG41 *+ M. oleifera + M. incognita*
T8	*M. incognita* only
T9	Healthy Control

After 45 days, growth parameters (plant height, root/shoot length, fresh/dry weights) and infection parameters (root gall rating, eggs per root, J2/100 cc soil, nematode reproduction factor) were assessed (Mukhtar et al., 2021).

### 2.7 Detection of volatile organic compounds from rhizobacteria

*Bacilllus australimaris* LWD73 was cultured overnight at 37°C in LB broth. The bacterial culture was adjusted to an optical density (OD) of 0.132 at 600 nm (0.5 McFarland units) using a spectrophotometer. A 5 mL aliquot was transferred to a headspace (HS) vial, and a Solid Phase Microextraction (SPME) fiber was inserted. The HS vial was incubated in a water bath at 60°C for 60 min. After incubation, the SPME fiber was immediately exposed to the GC injection port for 28 min. Each treatment was replicated nine times (Tait et al., 2014).

### 2.8 Compound identification from plant extracts

Nematicidal compounds in aqueous *M. oleifera* extracts were identified using Fourier Transform Infrared Spectroscopy (FTIR) (Elzey et al., 2016) and Gas Chromatography-Mass Spectrometry (GC-MS) (Ahmed et al., 2024).

### 2.9 Statistical analysis

Data on plant growth and infection parameters were analyzed using one-way analysis of variance (ANOVA) under a completely randomized design (CRD). Comparisons of means were conducted using Tukey’s Honestly Significant Difference (HSD) test. Statistical analyses were performed using GenStat software (12^th^ Edition, Version 12.1.0.3278, www.vsni.co.uk), at a significance level of p < 0.05.

## 3 Results

### 3.1 Molecular characterization of rhizobacteria

**Figure 1:**
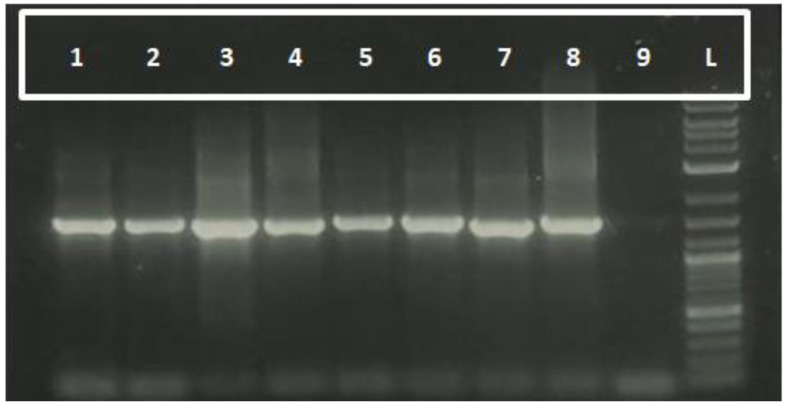
Molecular validation of rhizobacterial isolates using 16S rRNA Primers. Lane 1–8 represents the rhizobacterial isolates, 9 as control and L represents 1kb Ladder.

The molecular characterization of rhizobacterial isolates was conducted using the 16S rRNA gene region, amplified with universal primers 27F and 1492R. The amplification successfully yielded amplicons of approximately 1500 base pairs, as visualized through gel electrophoresis (**Figure 1**). This fragment size corresponds to the expected region of the 16S rRNA gene, which is widely used for bacterial identification and phylogenetic studies.

The sequencing data obtained from the amplified products were compared against the National Center for Biotechnology Information (NCBI) database using the BLAST algorithm to confirm the identity of the isolates at the species level. The rhizobacterial isolates, along with their respective GenBank accession numbers, are listed in **Table 2**. The high sequence similarity (>99%) with reference sequences in the database validated the taxonomic classification of the isolates.

**Table 2:** Identity and accession numbers of rhizobacteria.

Sr. No.	Isolate	Rhizobacteria Identified as	Accession No.
1	W2	*Bacillus australimaris* strain LWD73	OQ366704
2	W13	*B. cereus* strain HR001	OQ372951
3	W44	*B. thuringiensis* strain WAG41	OQ370579

### 3.2 Effect of rhizobacteria and plant extracts on tomato growth

The effect of different treatments of *M. oleifera*, both individually and in combination, was evaluated against *M. incognita* in terms of plant growth parameters (plant height, root length, fresh shoot weight, dry shoot weight, fresh root weight, dry root weight) and *M. incognita* infection parameters (root gall ratings, eggs per root system, J2/60 cc soil, and reproduction factor).

**Figure 2:**
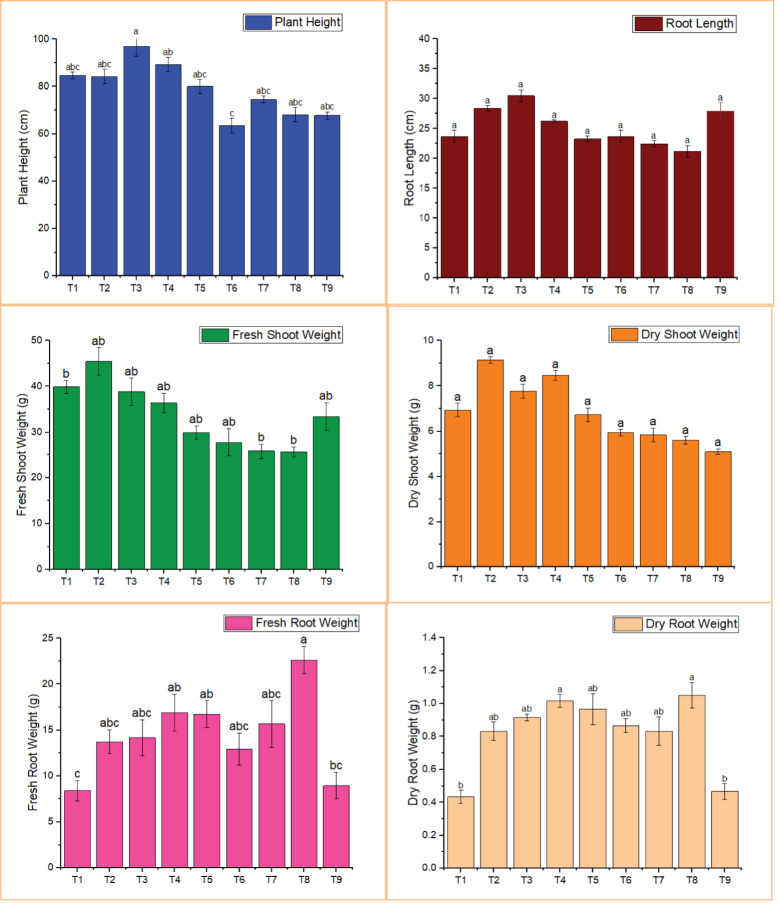
Effect of M. oleifera and rhizobacteria on the growth parameters of tomato. T1, *M. oleifera* + *M. incognita*; T2, *B. australimaris* LWD73 + *M. incognita*; T3, *B. australimaris* LWD73 + *M. oleifera* + *M. incognita*; T4, *B. cereus* HR001 + *M. incognita*; T5, *B. cereus* HR001 + *M. oleifera* + *M. incognita*; T6, *B. thuringiensis* WAG41 + *M. incognita*; T7, *B. thuringiensis* WAG41 + *M. oleifera* + *M. incognita*; T8, *M. incognita* only and T9, Healthy Control.

Plant height and root length were higher in nearly all treatments compared to the nematode control, but only treatment 3 differed significantly (**Figure 2**). The highest plant height (96.94 cm) and root length (30.48 cm) were recorded in T3 (BA-LWD73 + *M. oleifera*), whereas the lowest values (63.50 cm and 22.44 cm, respectively) were observed in T7 (BT-WAG41 + *M. oleifera*).

For fresh shoot weight and dry shoot weight, the highest values were recorded in T2, which involved the single treatment of BA-LWD73, with fresh and dry shoot weights of 45.43 g and 9.13 g, respectively. The lowest fresh shoot weight (25.80 g) was observed in T7 (BT-WAG41 + *M. oleifera*), while the lowest dry shoot weight (5.93 g) was recorded in both T6 and T7 (BT-WAG41 alone and in combination with *M. oleifera*) (**Figure 2**).

Fresh and dry root weights were lower in all treatments, significantly so in T1 and T9, compared to the nematode control. The lowest fresh root weight (8.40 g) was recorded in T1, which received the single treatment of *M. oleifera*. The lowest dry root weight (5.83 g) was observed in T7 (BT-WAG41 + *M. oleifera*) (**Figure 2**).

### 3.3 Effect of rhizobacteria and plant extracts on *M. incognita* infection

**Figure 3:**
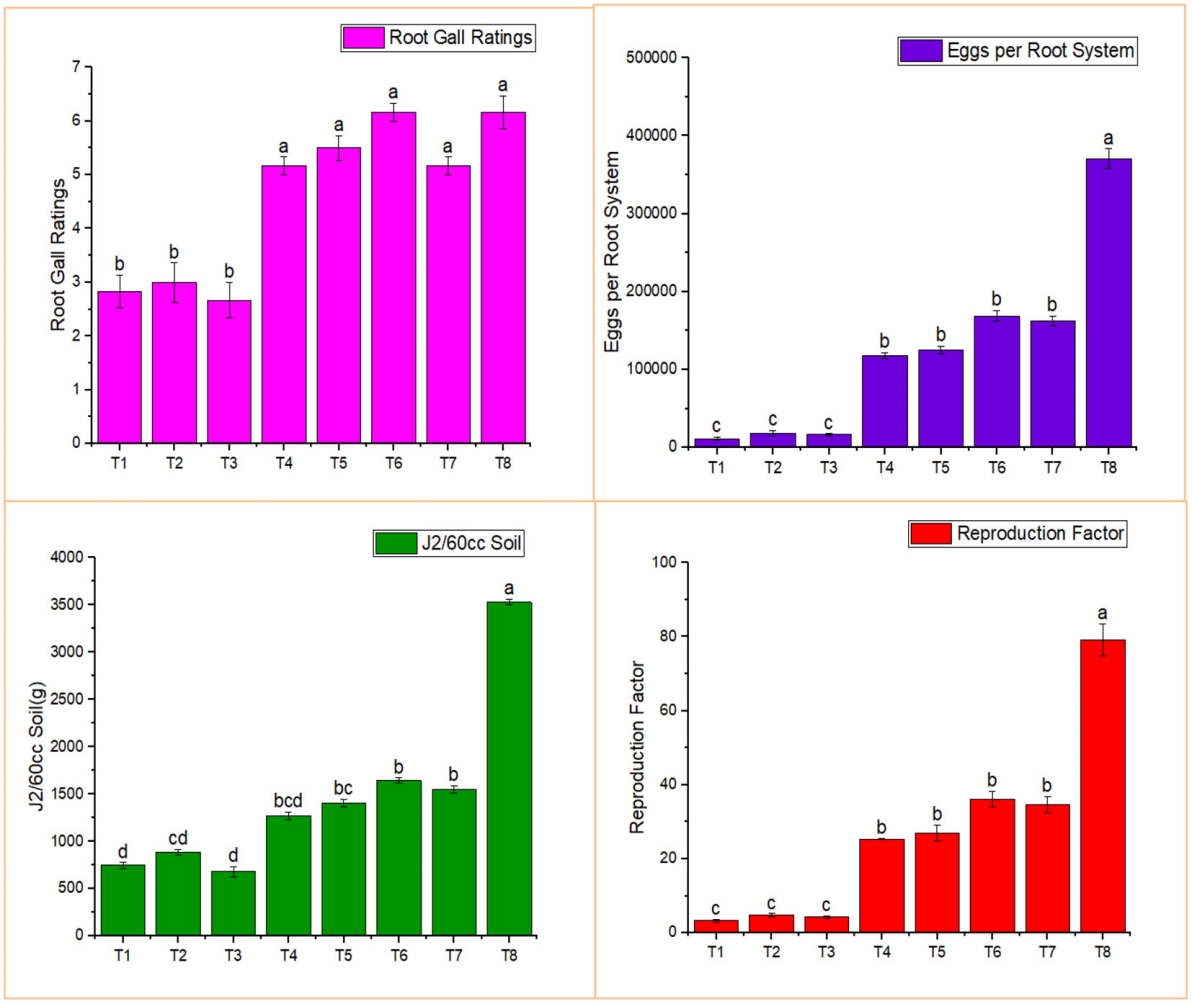
Effect of *M. oleifera* and rhizobacteria on infection parameters of *M. incognita*. T1, *M. oleifera* + *M. incognita*; T2, *B. australimaris* LWD73 + *M. incognita*; T3, *B. australimaris* LWD73 + *M. oleifera* + *M. incognita*; T4, *B. cereus* HR001 + *M. incognita*; T5, *B. cereus* HR001 + *M. oleifera* + *M. incognita*; T6, *B. thuringiensis* WAG41 + *M. incognita*; T7, *B. thuringiensis* WAG41 + *M. oleifera* + *M. incognita*; T8, *M. incognita* only and T9, Healthy Control.

Root gall ratings were lower in most treatments, significantly so in treatments T1-T3, compared to the positive control (**Figure 3**). The lowest root gall rating (2.67) was observed in T3, which consisted of the combined treatment of BA-LWD73 and *M. oleifera*, whereas T8 (*M. incognita* only, control) exhibited the highest root gall rating.

The single and combined applications of *M. oleifera* and BA-LWD73 resulted in 11,500, 18,000, and 16,500 eggs per root system, respectively. The lowest number of J2 was recovered from the single and combined applications of *M. oleifera* and BA-LWD73, with values of 746.83, 884.33, and 680 J2 per 60 cm³ of soil, respectively. Similarly, the lowest reproduction factors were recorded in the single and combined applications of *M. oleifera* and BA-LWD73, with values of 3.35, 4.84, and 4.25, respectively (**Figure 3**).

### 3.4 FTIR and GC-MS analysis of aqueous extracts of *M. oleifera*

**Figure 4:**
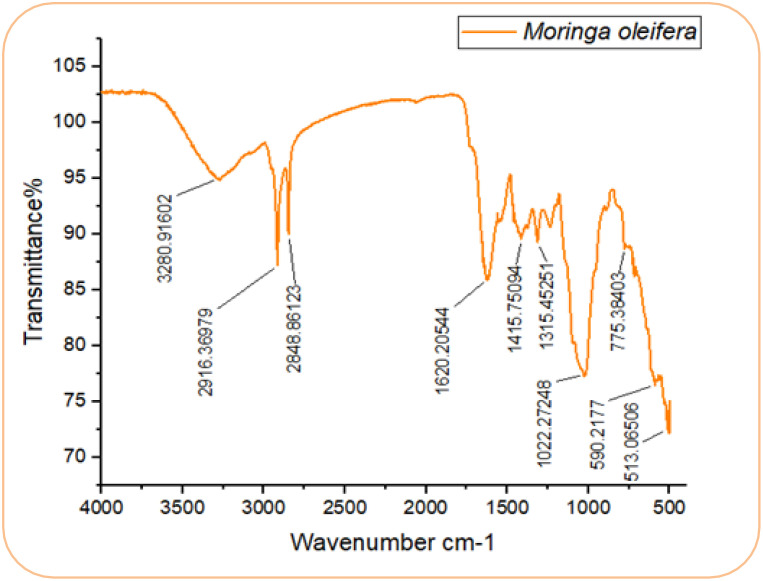
FTIR Spectra of *M. oleifera* leaves.

The FTIR spectra of *M. oleifera* are shown in **Figure 4**. The first and second peaks, observed at wavelengths 2916.36 cm^-^¹ and 2848.86 cm^-^¹, correspond to strong and broad stretching vibrations of hydroxyl groups, predominantly associated with alcohols. The third peak, at 1620.20 cm^-^¹, exhibited medium stretching vibrations, primarily indicating the presence of a C=C functional group, characteristic of alkenes. The fourth peak, at 1415.53 cm^-^¹, showed strong stretching vibrations attributed to S=O bonds, suggesting the presence of sulfates. The fifth peak, ranging from 1315.45 cm^-^¹ to 1022.27 cm^-^¹, exhibited strong stretching vibrations corresponding to C-O functional groups, predominantly signifying tertiary alcohols. The final peak, observed at 513.06 cm^-^¹, was characteristic of halo compounds.

The GC-MS chromatogram of the aqueous extract of *M. oleifera* revealed six distinct peaks, corresponding to six phytocompounds identified through comparison with the NIST library: 4(1H)-Pyrimidinone (3.02%), 2,6-Dimethyl acetic acid [(aminocarbonyl)amino]oxo (3.01%), Maltol (9.58%), 4H-Pyran-4-one, 2,3-dihydro-3,5-dihydroxy-6-methyl (10.30%), 5-Hydroxymethylfurfural (71.76%), and 4(1H)-Pyrimidinone, 2-(methylthio) (2.32%) (**Table 3**). These phytocompounds are reported to exhibit antimicrobial and nematicidal properties.

**Table 3:** Compounds identified in aqueous extract of *M. oleifera* L. by GC-MS analysis.

Peak No.	Compound Name	Molecular Formula	Molecular Weight (g/mol)	Retention Time (Min)	Peak Area (%)
1	4(1H)-Pyrimidinone, 2,6-dimethyl	C_24_H_30_N_2_O	362.5	5.310	3.02
2	Acetic acid, [(aminocarbonyl)amino]oxo	C_3_H_4_N_2_O_4_	132.08	5.561	3.01
3	Maltol	C_6_H_6_O_3_	126.11	5.705	9.58
4	4H-Pyran-4-one, 2,3-dihydro-3,5-dihydroxy-6-methyl	C_6_H_8_O_4_	144.12	6.155	10.30
5	5-Hydroxymethylfurfural	C_6_H_6_O_3_	126.11	7.636	71.76
6	4(1H)-Pyrimidinone, 2-(methylthio)-	C_5_H_6_N_2_OS	142.18	11.364	2.32

### 3.5 Detection of VOCs using SPME-GC-MS

**Figure 5:**
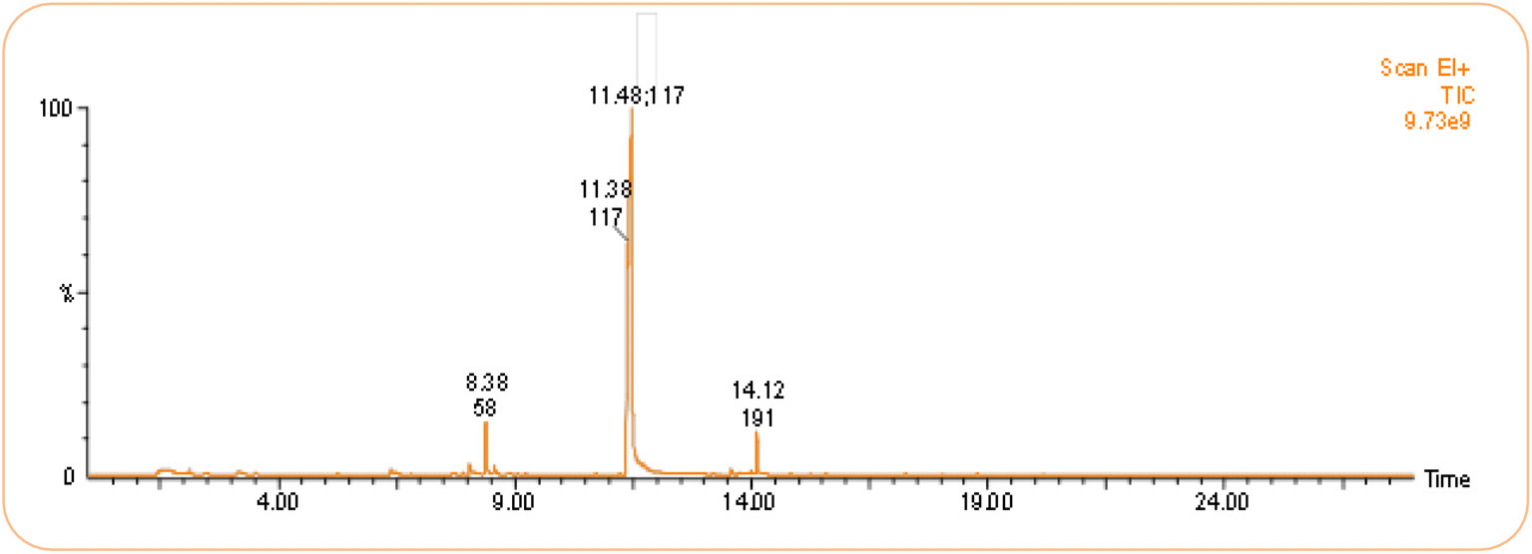
HS-SPME GC-MS chromatogram profiles of VOCs emitted from *B*. *australimaris* LWD-73.

Volatile organic compounds (VOCs) produced by *B. australimaris* strain LWD-73 were identified based on GC-MS analysis and comparison with the NIST library (**Figure 5**, **Table 4**). Four peaks were detected, corresponding to 2-Nonanone (6.54%), 1H-Indole (87.46%), Tetradecanol (0.99%), and 9-Hexadecenoic acid (5.02%).

**Table 4:** VOCs produced by *B. australimaris* LWD-73 detected by SPME-GC.

No.	Compound	Molecular Formula	Retention Time (min)	Molecular Weight (g/mol)	Peak Area	Percent
1	2-Nonanone	C_9_H_18_O	8.35	142.24	215618130	6.54
2	1H-Indole	C_8_H_7_N	11.50	117.15	2882910162	87.46
3	Tetradecanol	C_14_H_30_O	13.73	214.38	32564023	0.99
4	9-Hexadecenoic acid	C_16_H_32_O_2_	19.00	256.42	165322864	5.02

## 4 Discussion

*M. incognita* is one of the most economically important plant-parasitic nematodes worldwide, causing significant yield losses in a wide range of crops (Mukhtar and Hussain, 2019). It is highly prevalent in tropical and subtropical regions making it a major threat to agricultural productivity (Mukhtar and Kayani, 2019, 2020). Its widespread prevalence necessitates effective management strategies to mitigate its impact on agricultural productivity and food security.

In this study, the aqueous extract of *M. oleifera*, both alone and in combination with rhizobacterial strains, effectively reduced *M. incognita* populations and enhanced plant growth. Treatments combining *M. oleifera* with *B. australimaris* strain LWD-73 demonstrated superior performance compared to other treatments, significantly reducing nematode infection and promoting plant vigor.

Numerous plant species possess nematicidal constituents that suppress nematode populations and improve plant health (Mukhtar et al., 2013b). All parts of *M. oleifera* can be utilized as biopesticides due to their ability to suppress pathogens and enhance crop health (Yaseen and Hájos, 2020).

*M. oleifera* contains diverse bioactive compounds, including aldehydes, flavonoids, alcohols, phenols, and terpenoids, which may act individually or synergistically to affect nematodes. These compounds disrupt nematode feeding and reproduction, inhibit egg hatching, and exhibit juvenile toxicity. However, the precise mechanisms underlying their nematicidal activity remain unclear (Mukhtar et al., 2013b; Yaseen and Hájos, 2020).

Microbes also play a pivotal role in managing polyphagous root-knot nematodes across various crops and soil conditions (Mukhtar, 2018). They enhance plant health, induce systemic resistance, and help mitigate a wide range of biotic stresses. Rhizobacteria are particularly valuable for their contributions to nutrient uptake, phytohormone production, heavy metal stress mitigation, and increased crop yields (Groover et al., 2020; Bhat et al., 2023). Certain bacterial volatile organic compounds (VOCs), such as dimethyl disulfide, 2-nonanone, 1H-indole, tetradecanol, and 9-hexadecenoic acid, exhibit strong nematicidal activity against root-knot nematodes (Bhattacharyya et al., 2015; Agisha et al., 2019; Bhat et al., 2023).

FTIR and GC-MS analyses of *M. oleifera* extracts confirmed the presence of flavonoids, alcohols, and phenols, which are known for their antimicrobial and nematicidal properties. The nematicidal activity of *M. oleifera* was positively correlated with the abundance of these phytochemicals (Alam and Nuby, 2022; Anekwe et al., 2023).

Among the rhizobacteria tested, *B. australimaris* strain LWD-73 exhibited the most potent effects against *M. incognita*. SPME-GC-MS analysis identified key volatile organic compounds produced by *B. australimaris*, including 2-nonanone, 9-hexadecenoic acid, tetradecanol, and 1H-indole, all of which possess strong antimicrobial, antifungal, and nematicidal properties. For instance, 2-nonanone demonstrates significant nematicidal activity by inhibiting egg hatching, disrupting feeding, and reducing nematode populations, thereby limiting root damage over time (Huang et al., 2010; Cheng et al., 2017). Moreover, this compound exhibits antifungal activity against pathogens such as *Verticillium longisporum* and *Botrytis cinerea* (Rybakova et al., 2017).

Indole, another prominent VOC, interferes with nematode egg-laying and survival, induces oxidative stress at high concentrations, and triggers methuosis at low concentrations, ultimately leading to nematode mortality (Bommarius et al., 2013; Lee et al., 2017). Moreover, indole functions as a key chemical signal that promotes plant growth and influences auxin signaling. For example, indole emitted by *Escherichia coli* has been shown to enhance the root architecture of *Arabidopsis thaliana* (Bhattacharyya et al., 2015; Elzey et al., 2016; Ahmed et al., 2024).

9-Hexadecenoic acid (palmitoleic acid) exhibits nematicidal activity by inducing toxicity in larvae and eggs, acting as a repellent, and interfering with nematode feeding and reproduction. It may also stimulate plant defenses and influence soil microbial communities, presenting a promising approach to integrated nematode management (Cordovez et al., 2017).

Tetradecanol, emitted by *Paenibacillus polymyxa* strain J2-4, has demonstrated strong fumigant activity against *M. incognita*, further emphasizing the potential of volatile organic compounds in nematode management strategies (Song et al., 2024). These findings highlight the significance of plant-derived phytochemicals and microbial VOCs as sustainable tools for controlling nematodes and improving crop health.

## 5 Conclusions

The present study identified a novel bacterial strain, *Bacillus australimaris* strain LWD-73, with significant nematicidal activity, offering potential for the effective management of *Meloidogyne incognita*. The integration of this bacterial strain with moringa leaf extract demonstrated compatibility. Both moringa and the selected rhizobacteria are rich in phytochemicals with potent nematicidal and antimicrobial properties. These bioactive compounds provide promising solutions for nematode management and can be incorporated into integrated pest management (IPM) strategies. Small-scale vegetable growers can benefit from using aqueous moringa leaf extracts as a cost-effective and accessible method for nematode control, ultimately improving the health and productivity of vegetable crops.

## Data Availability

The raw data supporting the conclusions of this article will be made available by the authors, without undue reservation.
